# Congenital hepatic cyst with intracystic hemorrhage: Two rare cases reports in the Chinese Han population

**DOI:** 10.1097/MD.0000000000005161

**Published:** 2016-10-21

**Authors:** Qingqiang Ni, Minfeng Zhang, Cheng Yang, Wenchang Cai, Qian Zhao, Weifeng Shen, Jiamei Yang

**Affiliations:** a Medical College of Soochow University, Suzhou, Jiangsu, China; b Department of Special Treatment and Liver Transplantation, Eastern Hepatobiliary Surgery Hospital, Second Military Medical University, Shanghai, China; c Department of Pathology, Eastern Hepatobiliary Surgery Hospital, Second Military Medical University, Shanghai, China.

**Keywords:** congenital hepatic cyst, intracystic hemorrhage, liver resection

## Abstract

**Introduction:**

Fast-growing congenital hepatic cysts with intracystic hemorrhage are rare in clinical practice. Additionally, the clinical manifestations of and laboratory and imaging findings for this condition are often nonspecific and are particularly difficult to differentiate from those of hepatobiliary cystadenoma and cystadenocarcinoma, thus posing great challenges for diagnosis and treatment. The 2 case reports presented here aim to analyze the diagnosis and treatment of 2 rare cases of congenital hepatic cysts with intracystic hemorrhage in the Chinese Han population to provide an important reference for the clinical diagnosis and treatment of this condition.

**Diagnoses:**

These 2 case reports present 2 rare cases of congenital hepatic cysts with intracystic hemorrhage. Case 1 involved a 31-year-old patient with a very large, fast-growing hepatic cyst with intracystic hemorrhage and elevated carbohydrate antigen 199. Case 2 involved a patient with intense, paroxysmal right upper abdominal pain; computed tomography suggested a hepatic cyst with intracystic hemorrhage and possibly hepatobiliary cystadenoma.

**Outcomes:**

Both patients underwent liver resection. Postoperative follow-up showed that for both patients, the symptoms improved, the laboratory findings returned to normal levels, and the surgical outcomes were satisfactory.

**Conclusion:**

Liver resection is an ideal treatment for patients with congenital hepatic cysts with intracystic hemorrhage, and especially those with fast-growing, symptomatic hepatic cysts or hepatic cysts that are difficult to differentiate from hepatobiliary cystadenoma and cystadenocarcinoma.

## 1. Introduction

Generally speaking, “hepatic cyst” refers to various hepatic cystic diseases, which may be parasitic or nonparasitic, depending on the cause. Parasitic hepatic cysts are mainly observed in hydatid liver disease, whereas nonparasitic hepatic cysts may be congenital, inflammatory, traumatic, or neoplastic. Congenital hepatic cysts are the most common, so the term “hepatic cyst” usually refers to these cysts, also known as simple hepatic cysts, which is a condition that may be related to congenital biliary developmental aberrations.^[1]^ Congenital hepatic cysts may or may not be solitary. Hepatic cysts usually have a single compartment with a thin wall and clear intracystic fluid and without local thickening, nodules, or septa.

The incidence of congenital hepatic cysts is approximately 2.5%, and these are more common in women and tend to occur in the right lobe.^[2]^ Congenital hepatic cysts grow slowly, and most such cysts are asymptomatic and are detected during routine physical check-up. Congenital hepatic cysts are easy to diagnose and usually require no specific treatment except in cases of complications, such as intracystic hemorrhage, infection, cyst rupture, jaundice, or portal hypertension. Cases of fast-growing congenital hepatic cysts with intracystic hemorrhage are rare in clinical practice, accounting for approximately 10% of all cases of congenital hepatic cysts.^[3]^ The clinical manifestations and laboratory and imaging findings are often nonspecific and are particularly difficult to differentiate from those of hepatobiliary cystadenoma and cystadenocarcinoma, posing great challenges for diagnosis and treatment.^[4,5]^

Recently, our center admitted 2 Chinese Han patients with rare cases of congenital hepatic cysts with intracystic hemorrhage and successfully performed liver resection in these patients. The 2 case reports below aim to provide an important reference for the clinical diagnosis and treatment of hepatic cysts with intracystic hemorrhage.

## 2. Cases

### 2.1. Case 1

A 31-year-old female Chinese Han patient was admitted to the hospital “6 years after surgery for hepatic cyst rupture, showing abdominal distension with yellowing of the skin and sclera for 1 month.” Six years earlier, the patient had sought medical attention for “abdominal pain after abdominal impact.” Examination suggested “liver rupture, ascites,” and laparotomy showed “hepatic cyst rupture”; hence, the patient underwent “hepatic cyst resection,” and postoperative pathology suggested a “hepatic cyst.” During the operation, the surgeon found 2 additional “hepatic cysts” of approximately 2 to 3 cm in size but decided to leave them untouched. One month before presentation, the patient had developed abdominal distension with yellowing of the skin and sclera. Computed tomography (CT) performed at a local hospital suggested an extremely large hepatic cyst, and magnetic resonance imaging (MRI) suggested possibly cancerous hepatic cystadenoma.

**Figure 1:**
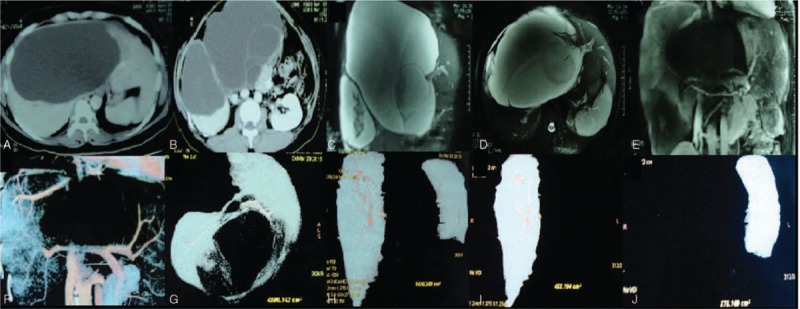
Imaging examination. (A, B) CT showed 2 masses (approximately 13.2 × 19.9 cm and 8.3 × 10.4 cm in size) of slightly low density. Septa were visible in the larger cyst, with enhancement of the cystic wall and septa and no significant enhancement inside the cyst. (C, D) MRCP showed dilation of the left intrahepatic bile ducts (possibly related to compression of the hilar bile ducts) and unclear extrahepatic bile ducts. (E) MRA showed compression and displacement of the hilar vessels, clear images of the trunk and branches of the portal vein, and compression and displacement of the hepatic veins. (F) CTA showed compression and displacement of the hilar vessels, clear images of the trunk and branches of the portal vein, a clear right hepatic vein (compressed and displaced), and an unclear left hepatic vein. (G, H, I, J) CT measurement showed that the overall liver volume was 4590 mL, and the FLV was 1030 mL: the FLV of the right liver was 455 mL, and the FLV of the left liver was 575 mL. CT = computed tomography, CTA = computed tomographic arteriography, FLV = future liver volume, MRA = magnetic resonance angiography.

The patient visited our hospital for further examination. Liver function tests showed total bilirubin 30.2 μmol/L, direct bilirubin 22.4 μmol/L, total bile acid 43.5 μmol/L, albumin 37 g/L, prealbumin 92 mg/L, and alanine aminotransferase 83 U/L. Hepatitis B surface antigen and antihepatitis C antibodies were negative. Tests for tumor markers showed that alpha-fetoprotein and carcinoembryonic antigen were negative. The carbohydrate antigen 199 (CA19-9) level was 298 U/mL. CT showed 2 masses (approximately 13.2 × 19.9 cm and 8.3 × 10.4 cm in size) of slightly low density. Septa were visible in the larger cyst, with enhancement of the cystic wall and septa and no significant enhancement inside the cyst (Fig. 1A and B). CT indicated multiple hepatic cystic lesions, which were possibly cystadenoma. Magnetic resonance cholangiopancreatography (MRCP) showed dilation of the left intrahepatic bile ducts (possibly related to compression of the hilar bile ducts) and unclear extrahepatic bile ducts (Fig. 1C and D), indicating multiple hepatic cystic lesions, which were possibly cystadenoma. Liver magnetic resonance angiography showed compression and displacement of the hilar vessels, clear images of the trunk and branches of the portal vein, and compression and displacement of the hepatic veins (Fig. 1E), indicating a huge hepatic cystic lesion. Liver computed tomographic arteriography showed compression and displacement of the hilar vessels, clear images of the trunk and branches of the portal vein, a clear right hepatic vein (compressed and displaced), and an unclear left hepatic vein (Fig. 1F). CT measurement showed that the overall liver volume was 4590 mL, the functional liver volume (FLV) was 1030 mL, the FLV of the right liver was 455 mL, and the FLV of the left liver was 575 mL (Fig. 1G–J). As the calculated standard liver volume (SLV) was 1231 mL and the FLV of the right liver was 455 mL, the SLV-to-FLV ratio (SFLVR; FLV/SLV%) for the right liver was 37.0%, and given that the FLV of the left liver was 575 mL, the SFLVR for the right liver was 46.7%.

**Figure 2:**
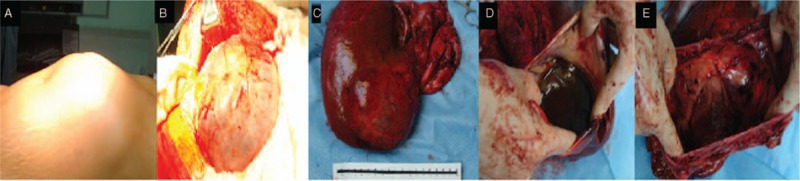
Intraoperative exploration and gross examination of the surgical specimen. (A, B) Intraoperative exploration showed a huge cystic mass of approximately 25 × 20 cm in size in the middle lobe, extending down to the pelvis and adhering to the gallbladder and the first porta hepatis. Moreover, another cystic mass, approximately 10 × 10 cm in size, was present in the lower right lobe immediately adjacent to the huge cystic mass. (C, D, E) Gross examination of the surgical specimen showed that the specimen was 22.3 × 16 × 8.4 cm in size; the cystic cavity was 20 × 11 cm in size (as observed based on the specimen surface and sections); the cavity was filled with brown turbid liquid; the interior cystic wall was rough and 0.2 to 0.7 cm thick, with a small amount of bleeding in part of the wall; and no apparent cirrhosis was observed in the remaining liver tissue.

**Figure 3:**
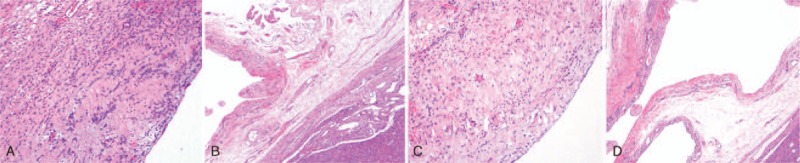
Histopathological examination. (A, B) Microscopic examination showed that the cystic wall was covered with a single layer of squamous epithelial cells, with hyperplasia of the fibrous tissue (especially elastic fibers) beneath the epithelial cells, infiltration of a small number of inflammatory cells (hematoxylin and eosin stain: A, magnification × 100; B, magnification × 40). (C, D) Microscopic examination showed that the cystic wall was covered with a single layer of squamous epithelial cells, with hyperplasia of the fibrous tissue underneath the epithelial cells, infiltration of inflammatory cells. (Hematoxylin and eosin stain: C, magnification × 100; D, magnification × 40).

The patient had no history of exposure to pastoral areas and no family history of cancer or genetic diseases. The patient was scheduled for resection of the 3 right lobes and the gallbladder. Intraoperative exploration showed a huge cystic mass of approximately 25 × 20 cm in size in the middle lobe, extending down to the pelvis and adhering to the gallbladder and the first porta hepatis. Moreover, another cystic mass, which was approximately 10 × 10 cm in size, was present in the lower right lobe, immediately adjacent to the huge cystic mass (Fig. 2A and B). Due to its size, the huge cystic mass in the middle lobe affected the operation; thus, the mass was punctured, and approximately 3400 mL of brown turbid liquid with a foul smell was withdrawn. Both the lateral left lobe and the posterior right upper lobe were enlarged, with a palpable right hepatic vein and an impalpable left hepatic vein. Resection of the 3 right lobes was planned. During the operation, the right hilum was blocked for 16 minutes, the first porta hepatis was blocked for 3 minutes, and the inferior vena cava was blocked for 29 minutes. Blood loss was approximately 2000 mL, and intraoperative transfusion included 1600 mL of red blood cells and 800 mL of plasma. According to the postoperative pathology report, gross examination of the surgical specimen showed that the specimen was 22.3 × 16 × 8.4 cm in size; the cystic cavity was 20 × 11 cm in size (as observed based on the specimen surface and sections); the cavity was filled with brown turbid liquid; the interior cystic wall was rough and 0.2 to 0.7 cm thick, with a small amount of bleeding in part of the wall; and no apparent cirrhosis was observed in the remaining liver tissue (Fig. 2C–E). Microscopic examination showed that the cystic wall was covered with a single layer of squamous epithelial cells, with hyperplasia of the fibrous tissue (especially elastic fibers) beneath the epithelial cells, infiltration of a small number of inflammatory cells (Fig. 3A and B). Pathological examination indicated liver cysts. After the operation, the patient recovered well, and cystic fluid culture showed no bacterial growth. CA19-9 returned to normal at 1 month after the operation, and the bile leakage that occurred after the operation self-resolved in 3 weeks.

### 2.2. Case 2

**Figure 4:**
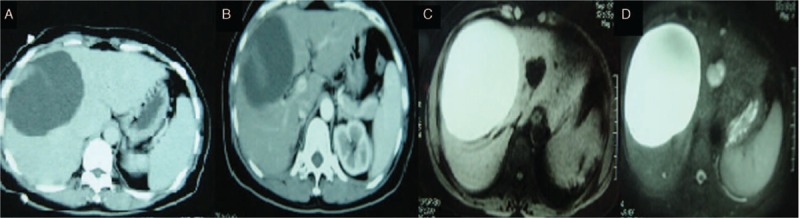
Imaging examination and postoperative specimen. (A, B) CT showed a right-lobe mass of approximately 11 × 8 cm in size. Enhanced CT showed a cystic mass at the edge of the liver parenchyma, with uneven enhancement during the arterial phase. The density of the lesion was lower than that of the surrounding normal liver parenchyma during the portal and delayed phases. (C, D) MRCP showed a mass with high T1 and T2 signals, approximately 10 × 13 cm in size, with multiple nodules on the wall in the right anterior lobe. CT = computed tomography, MRCP = magnetic resonance cholangiopancreatography.

Two months before presentation, a 45-year-old female Chinese Han patient had experienced right upper abdominal distension and intense pain of unknown origin; the symptoms lasted several minutes and then resolved without treatment. An ultrasound B-mode scan showed a cystic mass in the right lobe of approximately 11.5 × 8.6 cm in size, which was possibly a hepatic cyst. Liver CT suggested a right-lobe mass of approximately 11 × 8 cm in size. Enhanced CT showed a cystic mass at the edge of the liver parenchyma, with uneven enhancement during the arterial phase; the density of the lesion was lower than that of the surrounding normal liver parenchyma during the portal and delayed phases, indicating possible hepatic cystadenoma (Fig. 4A and B). MRCP showed a mass of approximately 10 × 13 cm in size with high T1 and T2 signals and with multiple nodules on the wall of the right anterior lobe (Fig. 4C and D). Laboratory tests were largely normal, and liver function was rated Child grade A; thus, the lesion was operable. During the operation, a cystic lesion of approximately 12 × 12 cm in size was observed in liver segments 5 and 8, directly adjacent to the gallbladder. Thus, resection of liver segments 5 and 8 and the gallbladder was planned. During the operation, the hilum was blocked for 25 minutes. Blood loss was approximately 500 mL, 1500 mL of fluid was infused, and no blood transfusion was given. The cystic fluid was brown and turbid. According to the postoperative pathology report, gross examination showed that the cystic wall-like tissue was 13.2 × 7 cm in size and that the interior cystic wall (0.1- to 0.4-cm thick) was smooth. Additionally, the gallbladder was 8.1 × 4.2 cm in size, the gallbladder wall was 0.1 to 0.2 cm in thickness, and the mucosa was rough. Microscopic examination showed that the cystic wall was covered with a single layer of squamous epithelial cells, with hyperplasia of the fibrous tissue underneath the epithelial cells, infiltration of inflammatory cells (Fig. 3C and D). Pathological examination suggested a hepatic cyst and chronic cholecystitis. The patient recovered well after the operation.

## 3. Discussion

In hepatic cysts, intracystic hemorrhage may be caused by excessive intracystic pressure and the ensuing necrosis of vascular epithelial tissue of the cystic wall.^[6]^ The cystic fluid is mahogany colored or bloody. For the 2 patients described here, the cystic fluid was brown and turbid; thus, the properties of the cystic fluid did not support intracystic hemorrhage. However, the patients had no fever, and cystic fluid culture showed no bacterial growth; thus, cystic infection was excluded. If communicated to the bile duct, cystic fluid should be light yellow, and thus, this possibility was also excluded. Based on imaging findings (see below) and the pathological process of hemoglobin destruction or absorption after intracystic hemorrhage, we made a diagnosis of a hepatic cyst with intracystic hemorrhage. We invite discussion of other reasonable diagnostic hypotheses that might explain the pathological changes observed in these 2 cases.

The clinical manifestations of hepatic cysts with intracystic hemorrhage are nonspecific, and patients with this condition may seek medical attention for a series of clinical manifestations and complications related to the size of the cyst, such as acute or chronic abdominal pain, a fast-growing mass during a regular check-up, and jaundice. The patient in Case 1 visited the hospital due to abdominal distension, mass, and yellowing of the skin and sclera. Fast-growing cysts must be differentiated from hepatobiliary cystadenoma and cystadenocarcinoma. The patient in Case 2 visited the hospital due to intense paroxysmal right upper abdominal pain, which should be differentiated from acute cholecystitis. In addition, a congenital hepatic cyst with intracystic hemorrhage should be differentiated from hydatid liver disease and liver abscess.

The imaging and laboratory findings for a hepatic cyst with intracystic hemorrhage are nonspecific. T1-weighted MRI images may show high signals for hemorrhagic cysts and low signals for nonhemorrhagic cysts, and T2-weighted images may show high signals for both hemorrhagic cysts and nonhemorrhagic cysts. CT usually shows low density for nonhemorrhagic cysts and may show high density for hemorrhagic cysts in the acute stage and low density or mixed density after the acute stage. Vilgrain et al^[3]^ reported clinical data for 4 patients with congenital hepatic cysts with intracystic hemorrhage, for whom MRI showed high signals on both T1 and T2 images, similar to what was observed in Case 2. Hagiwara et al reported 1 case of a cystic lesion, 8 cm in size, that was detected by an ultrasound B-mode scan. The cystic wall was thin and smooth, with scattered calcifications and small nodules. Enhanced CT and MRI showed mild enhancement, and MRI showed high T1 and T2 signals in the cystic fluid. The patient was considered to have a malignant tumor and underwent liver resection. During the operation, the cystic fluid was bloody, and postoperative pathological examination confirmed that the nodules were organized hematomas.^[7]^

Serum and cystic fluid CA19-9 levels may not contribute to distinguishing between congenital hepatic cysts with intracystic hemorrhage and cystadenoma or cystadenocarcinoma. In Case 1 in the present study, serum CA19-9 was significantly elevated (CA19-9 in the cystic fluid: NA), and in Case 2, serum CA19-9 was not high (CA19-9 in the cystic fluid: NA). Dinc et al^[8]^ reported 1 case of a huge hepatic cyst with elevated serum CA19-9; the authors believed that the elevated CA19-9 may have been related to compression of the surrounding organs by the huge hepatic cyst and excessive secretion by the epithelial tissue in the thickened cystic wall. Horsmans determined CA19-9 levels in the serum and the cystic fluid in 1 case of cystadenoma and 1 case of cystadenocarcinoma and found that both serum CA19-9 and CA19-9 in the cystic fluid were elevated in cystadenoma and cystadenocarcinoma, whereas only CA19-9 in the cystic fluid was elevated in a congenital hepatic cyst.^[9]^ CA19-9 may not contribute to the differential diagnosis of congenital hepatic cysts with intracystic hemorrhage.^[10]^ Choi et al^[11]^ showed that CA19-9 and carcinoembryonic antigen levels in the cystic fluid were of little significance for differentiating between biliary cystadenoma and a simple hepatic cyst. Bertino et al^[12,13]^ showed that serum CA19-9 levels were frequently elevated in chronic viral hepatitis and hepatic cirrhosis. Serum CA19-9 levels may be elevated due to compression of the surrounding organs by the huge hepatic cyst and the excessive secretion by epithelial tissue in the thickened cystic wall. Elevated cystic fluid CA19-9 levels may be detected in patients with elevated serum CA19-9 levels and intracystic hemorrhage, which may be caused by excessive intracystic pressure and ensuing necrosis of vascular epithelial tissue of the cystic wall. Thus, in Case 1, CA19-9 in the cystic fluid may be significantly elevated, and in Case 2, CA19-9 in the cystic fluid may be not high.

A congenital hepatic cyst with intracystic hemorrhage may be treated with a nonsurgical or surgical approach. Nonsurgical approaches mainly include percutaneous drainage, which is primarily used in confirmed cases in the elderly and in patients with a poor general condition. The relapse rate for nonsurgical approaches is relatively high; studies have reported that the relapse rate for percutaneous drainage is up to 100%.^[14]^ Surgical approaches include laparoscopy and open cyst fenestration, liver resection, and liver transplantation.^[15]^ Hotta et al^[16]^ and Poggi et al^[17]^ each reported 1 case of spontaneous rupture of a huge hepatic cyst. In the present study, the 2 patients had obvious symptoms and were at risk of cyst rupture. The cysts could have been hepatobiliary cystadenoma or a malignancy such as cystadenocarcinoma, and the cysts were large. Thus, the patients underwent open resection. After the operations, the patients recovered well, with satisfactory outcomes. Martin et al^[1]^ showed that no patient requires a second surgery after liver resection of a symptomatic hepatic cyst. We believe that liver resection should be considered if cystadenoma or cystadenocarcinoma cannot be ruled out and in cases with turbid cystic fluid if it is not clear whether the cyst communicates with the bile duct or whether intracystic hemorrhage or infection is present. Liver transplantation is indicated in patients with poor liver function who cannot tolerate surgery and in patients whose FLV is too small.

In Case 1, both cysts were large and located in the middle lobe, posing great challenges for liver resection. In addition, the patient had a history of surgery; before the operation, she had experienced liver damage (high total bilirubin and low prealbumin [possibly related to wasting and loss of appetite]), and her left hepatic vein was unclear on radiographic images. Due to these unfavorable surgical factors, it was extremely difficult to preserve her left hepatic lobe. In terms of preserving the FLV, cystectomy retained the maximum amount of liver tissue, which ensured postoperative recovery. However, the operation was associated with a long operation time, a large liver wound, and high blood loss, all of which increase surgical risk. In contrast, resection of 3 lobes simplified the operation. Thus, before the operation, an accurate assessment was performed, and the FLV was calculated to determine the surgical approach. According to Urata's method, the patient's body surface area (body weight [kg]^0.425^ × height [cm]^0.725^ × 0.007184 m^2^) was first calculated, after which the SLV was calculated (formula: [706.2 × body surface area (m^2^) + 2.4] mL) and the SFLVR was calculated.^[18]^ In Asian populations, the safety threshold for donors for live liver transplantation is 30% of the SLV.^[19]^ For Case 1, the FLV of the right liver was 455 mL, and the SFLVR was 37.0%, whereas the FLV of the left liver was 575 mL, and the SFLVR was 46.7%. Thus, either the left or the right liver could have been preserved, but it was obviously safer to preserve the left liver. During the operation, the 3 right lobes were resected while effectively preserving the FLV. The patient recovered well after the operation.

## 4. Conclusion

For congenital hepatic cysts with intracystic hemorrhage, in patients with a poor general condition and elderly patients with a confirmed preoperative diagnosis, nonsurgical approaches can be used. In addition, for fast-growing, symptomatic, and huge hepatic cysts as well as cysts that are difficult to differentiate from hepatobiliary cystadenoma and cystadenocarcinoma, liver resection may be an ideal treatment if the benefits and risks are thoroughly evaluated before the operation.
